# Questions on the Structure and Development of Human Teeth

**Published:** 1878-09

**Authors:** 


### BIBLIOGRAPHICAL.

*Questions on the Structure and Development of Human Teeth.* —By C. L. Ford, M. D., Professor of Anatomy and Physiology in the University of Michigan, Ann Arbor. Douglas & Co., publishers.

A pamphlet of 16 pages, embracing some 350 questions on the subject of Teething, the Alveolus, Vascular and Nervous supply of Teeth, their structure, etc., the bones, muscles, saliva, and comparative anatomy of teeth. Useful to the dental student as formulating his ideas of what he is to study. As the answers to these questions are not given, the questions themselves suggest fields of research to the student worthy of being in the hands of every student.

---

### MONTHLY SUMMARY.

